# Genome-wide association analysis of sucrose and alanine contents in edamame beans

**DOI:** 10.3389/fpls.2022.1086007

**Published:** 2023-02-03

**Authors:** Zhibo Wang, Dajun Yu, Gota Morota, Kshitiz Dhakal, William Singer, Nilanka Lord, Haibo Huang, Pengyin Chen, Leandro Mozzoni, Song Li, Bo Zhang

**Affiliations:** ^1^ School of Plant and Environmental Sciences, Virginia Tech, Blacksburg, VA, United States; ^2^ Department of Food Science and Technology, Virginia Tech, Blacksburg, VA, United States; ^3^ School of Animal Sciences, Virginia Tech, Blacksburg, VA, United States; ^4^ Fisher Delta Research Center, University of Missouri, Portageville, MO, United States; ^5^ Department of Crop, Soil, and Environmental Sciences, University of Arkansas, Fayetteville, AR, United States

**Keywords:** GWAS, edamame, sucrose, alanine, sensory

## Abstract

The sucrose and Alanine (Ala) content in edamame beans significantly impacts the sweetness flavor of edamame-derived products as an important attribute to consumers’ acceptance. Unlike grain-type soybeans, edamame beans are harvested as fresh beans at the R6 to R7 growth stages when beans are filled 80-90% of the pod capacity. The genetic basis of sucrose and Ala contents in fresh edamame beans may differ from those in dry seeds. To date, there is no report on the genetic basis of sucrose and Ala contents in the edamame beans. In this study, a genome-wide association study was conducted to identify single nucleotide polymorphisms (SNPs) related to sucrose and Ala levels in edamame beans using an association mapping panel of 189 edamame accessions genotyped with a SoySNP50K BeadChip. A total of 43 and 25 SNPs was associated with sucrose content and Ala content in the edamame beans, respectively. Four genes (Glyma.10g270800, Glyma.08g137500, Glyma.10g268500, and Glyma.18g193600) with known effects on the process of sucrose biosynthesis and 37 novel sucrose-related genes were characterized. Three genes (Gm17g070500, Glyma.14g201100 and Glyma.18g269600) with likely relevant effects in regulating Ala content and 22 novel Ala-related genes were identified. In addition, by summarizing the phenotypic data of edamame beans from three locations in two years, three PI accessions (PI 532469, PI 243551, and PI 407748) were selected as the high sucrose and high Ala parental lines for the perspective breeding of sweet edamame varieties. Thus, the beneficial alleles, candidate genes, and selected PI accessions identified in this study will be fundamental to develop edamame varieties with improved consumers’ acceptance, and eventually promote edamame production as a specialty crop in the United States.

## Introduction

Edamame, vegetable or edible soybean, is a nutritious food source of protein, isoflavones, dietary fibers, and vitamins (Mahoussi et al., 2020; Carneiro et al., 2021; Dhakal et al., 2021; Yu et al., 2022). Edamame has been cultivated and consumed in east Asian countries for centuries and documented edamame varieties are mainly originated from this area (Carneiro et al., 2021; Dhakal et al., 2021; Yu et al., 2022). In the past two decades, production and breeding of locally adapted edamame varieties have been reported in North America, and its consumption has increased (Wolfe et al., 2018). More attention has been paid to edamame as a potential alternative crop among growers and processors in the mid-Atlantic and Southeast United States (Carson et al., 2011; Ogles et al., 2016).

Typically, edamame is eaten as a snack or added to salads, stews and soups (Kumar et al., 2011; Carneiro et al., 2021). Sensory surveys of edamame have shown that the sweetness of edamame bean is positively correlated with consumer acceptance (Yu et al., 2022). Two components, sucrose and Ala, are the main contributors to the sweetness of edamame products (Hou et al., 2009; Yu et al., 2022). To promote domestic production and consumption of edamame, the varieties with improved seed sweetness are needed to match American consumer expectations for edamame products. Therefore, in addition to improving the nutritional content and agronomic properties of edamame, breeders must also consider edamame sensory attributes, such as appearance, flavor and texture, especially sweetness, to increase domestic market (Carneiro et al., 2020). Newly developed edamame variety with improved natural seed sweetness also helps reduce supplementation of added artificial sweeteners.

Soybean seeds typically contain about 35% carbohydrates on a dry matter basis, of which about 40% and 60% are soluble sugars and insoluble sugars, respectively (Sui et al., 2020). Soluble sugars usually consist of 5% sucrose, 1.5% raffinose, and 3% stachyose (Wang et al., 2014; Sui et al., 2020). Sucrose content varies widely among soybean accessions. The sucrose content in cultivated and wild soybean seeds is usually 4-6% and 3-4%, respectively (Sui et al., 2020). Numerous genetic studies on the inheritance of sucrose content in soybean seeds have been reported. Based on the recombinant inbred line (RIL) population crossed between V71-370 and PI 407162, 17 QTL associated with sucrose content were identified on chromosomes (Chr) 5, 7, 8, 13, 15, 19 and 20 (Maughan et al., 2000). Using RIL populations from the ‘Keunolkong’ × ‘Shinpaldalkong’ cross, six QTL associated with sucrose content were characterized on Chr 2, 11, 12, 16 and 19 (Kim et al., 2005). A major QTL on Chr 6 in the Sat_213-Satt643 interval was identified from two F_2_ populations, which could explain 76% of the observed genetic variation of sucrose content (Skoneczka et al., 2009). A sucrose QTL was reported on Chr 11 from an F_2_ population derived from V97-3000 × V99-5089 (Wang et al., 2014) while four QTL were identified on Chr 6, 8, 16 and 20 from a cross between *G. max* (Williams 82 (WM82)) and *G. soja* (PI 483460B) (Patil et al., 2018). Five SNPs located on Chr 4, 6, 7, 11, and 12 were detected to be associated with sucrose content in a genome-wide association study (GWAS) analysis of 178 diverse soybean accessions (Sui et al., 2020). In summary, there was a total of 362 SNPs and 31 bi-parental QTL (Quantitative Trait Locus) being identified to associate with soybean sucrose content. As another major sweetness contributor, the content of Ala also varies widely in soybean seeds, 15 - 23 mg g^-1^ dry seed (Qin et al., 2019). A GWAS reported 11 SNPs being related with Ala content in soybean seeds (Qin et al., 2019).

As the cost of genotyping has decreased and statistical methods have been improved, GWAS has become a popular method for understanding the genetic basis of complex traits, enabling more concise breeding of various crop species including soybean, cotton, wheat, rice, maize, and sorghum (Khan et al., 2018; Liu et al., 2018; Jiang et al., 2020; Sui et al., 2020; Gahlaut et al., 2021; Verma et al., 2022; Singer et al., 2022). GWAS is also complementary to QTL mapping and linkage analysis, where QTL analysis deals with the contribution of a locus to variation in continuous trait while GWAS studies the association between alleles and traits (Liu et al., 2018). In soybean, GWAS has been used to characterize the molecular basis of complex traits, as it significantly increases the range of phenotypic variation based on the accumulation of historical recombination events. Specifically, GWAS has been used for agronomic traits (Chang et al., 2018), seed quality traits including protein, oil, and fatty acid content (Zhang et al., 2019; Zhao et al., 2019), disease resistance (Hanson et al., 2018), and abiotic stress adaptation (Do et al., 2019). Advances in genome sequencing have accelerated the resolution and accuracy of the application of GWAS in soybean research (Li et al., 2015).

Because edamame beans are harvested when the seeds are immature, the chemical properties of edamame, especially the sucrose and Ala levels are different from those in dry soybean seeds. As a result, the genetic basis responsible for the sucrose and Ala contents between edamame and dry seeds might be different. The objective of this study was to investigate the genes associated with sucrose and Ala contents in edamame through a GWAS approach in order to better assist edamame breeding on sensory and flavor.

## Methods and materials

### Plant materials

A total of 189 collected edamame accessions (**Table S1**) were planted at Blacksburg (Virginia), Portageville (Missouri), and Fayetteville (Arkansas) in the years of 2018 and 2019. The seeds were sown in 3 m row and 0.75 m row spacing (with a seeding rate of approximately 70,000 plants per hectare). Field trials were performed with three to four replications in a randomized complete block design (RCBD). The edamame beans were harvested at R6-R7 stages when the bean was filled 80-90% of the pod capacity to quantify sucrose and Ala contents.

### Measurements of sucrose content and Ala content in edamame beans

Sucrose and Ala contents in edamame beans were measured using the procedures described by Yu et al. (2021) (Yu et al., 2021; Yu et al., 2022). Briefly, the freeze-dried beans were ground to powder to pass through a 500-μm sieve. Subsequently, 0.15 g of the powder was mixed with 1.5 mL of deionized (DI) water and then shaken for 2 hours at room temperature. The mixture was centrifuged at 13,500 g for 10 minutes, and 750 μL of supernatant was collected and shaken with an equal volume of acetonitrile for 10 minutes. With another centrifuge at 13,500 g for 10 minutes, 750 μL of supernatant was collected and filtered by a 0.20-μm membrane filter. The pass-through was measured for its sucrose and Ala contents by high performance liquid chromatography (HPLC).

Sucrose content was determined by HPLC with a refractive index detector (RID) (Agilent Technologies, Santa Clara, CA, USA). A Luna Omega 3 μm SUGAR column (150 × 4.6 mm, Phenomenex, Torrance, CA, USA) was applied for sugar separation at 40°C in which acetonitrile/water (75:25 v:v) was used as the mobile phase. The flow rate was 1 mL/min and the injection volume was 5 μL.

To determine the Ala content, HPLC was performed using online derivatization with o-Phthalaldehyde (OPA), and the Agilent AdvanceBio Amino Acid Analysis (AAA) C18 column with a 1200 diode array detector at λ = 338 nm. Mobile phase A contained 10 mM Na_2_HPO_4_, and 10 mM Na_2_B_4_O_7_ with pH 8.2 while mobile phase B mixed acetonitrile, methanol, and DI water (45:45:10, v: v: v). The flow rate was set at 1.5 mL/min with a gradient program for separation with details described in Yu et al. (2021). Each sample for sucrose and Ala measurements had two technical replicates which were averaged to account for biological and equipment variation.

### Population structure analysis

A discriminant analysis of principal components (DAPC) was conducted to assess the population structure of the panel by using the adegenet package in R (Jombart et al., 2010). A total of three principal components were retained to maximize cumulative variance and Bayesian information criterion (BIC) was used to identify an optimal number of clusters.

### Phenotypic adjustment using best linear unbiased estimator

Best linear unbiased estimates (BLUE) of genotypes were inferred while accounting for systematic effects. The BLUE values were obtained using the equation below,


$$y_{ijk}=\mu +r_{i}+b_{j}+g_{k}+e_{ijk}$$

Where *y_ijk_* is a vector of the of sucrose or Ala content for the *k*th genotype in the *i*th replication and the *j*th environment, *μ*is the grand mean, *r_i_* is the fixed effect of replication, *b_j_* is the fixed effect of environment (combined analysis only), *g_k_* is the fixed effect of genotype, and *e_ijk_* is the model residual.

### Genome-wide association analysis

The genetic signals associated with the sucrose content and Ala content in edamame beans were identified using a SoySNP50K iSelect BeadChip (Song et al., 2013). The association between 41,985 SNPs and traits was determined using a mixed linear model (MLM) implemented in the R package rrBLUP (Endelman, 2011). In total, we generated 10 phenotypic datasets for exploring the SNPs that associated with the sucrose and Ala contents. There are six datasets for six individual environments, Blacksburg in 2018 or 2019, Portageville in 2018 or 2019, and Fayetteville in 2018 or and 2019. The two years’ data for the same location were combined and displayed as: Blacksburg combined dataset, Portageville combined dataset, and Fayetteville combined dataset. At last, BLUE (Haslett and Puntanen, 2009) was introduced to adjust the phenotypic data scored across all locations and years.

A modified Šidák correction (*α_sid_* = 1 − (1 − *α*)^1/^ *^m^*) for multiple testing was used to derive a statistical significance threshold. The effective number of markers (*M*_eff_) was calculated to replace *m*, and thus, the adjusted significance threshold at *α* = 5% and the suggestive threshold at *α* = 10% were (*P*) > 3.01 and (*P*) > 2.70, respectively (Li and Ji, 2005). The R packages ggplot2 and dplyr were used to generate Manhattan and quantile-quantile (QQ) plots.

## Results

### Statistical and variation analysis of sucrose and Ala contents

**Figure 1:**
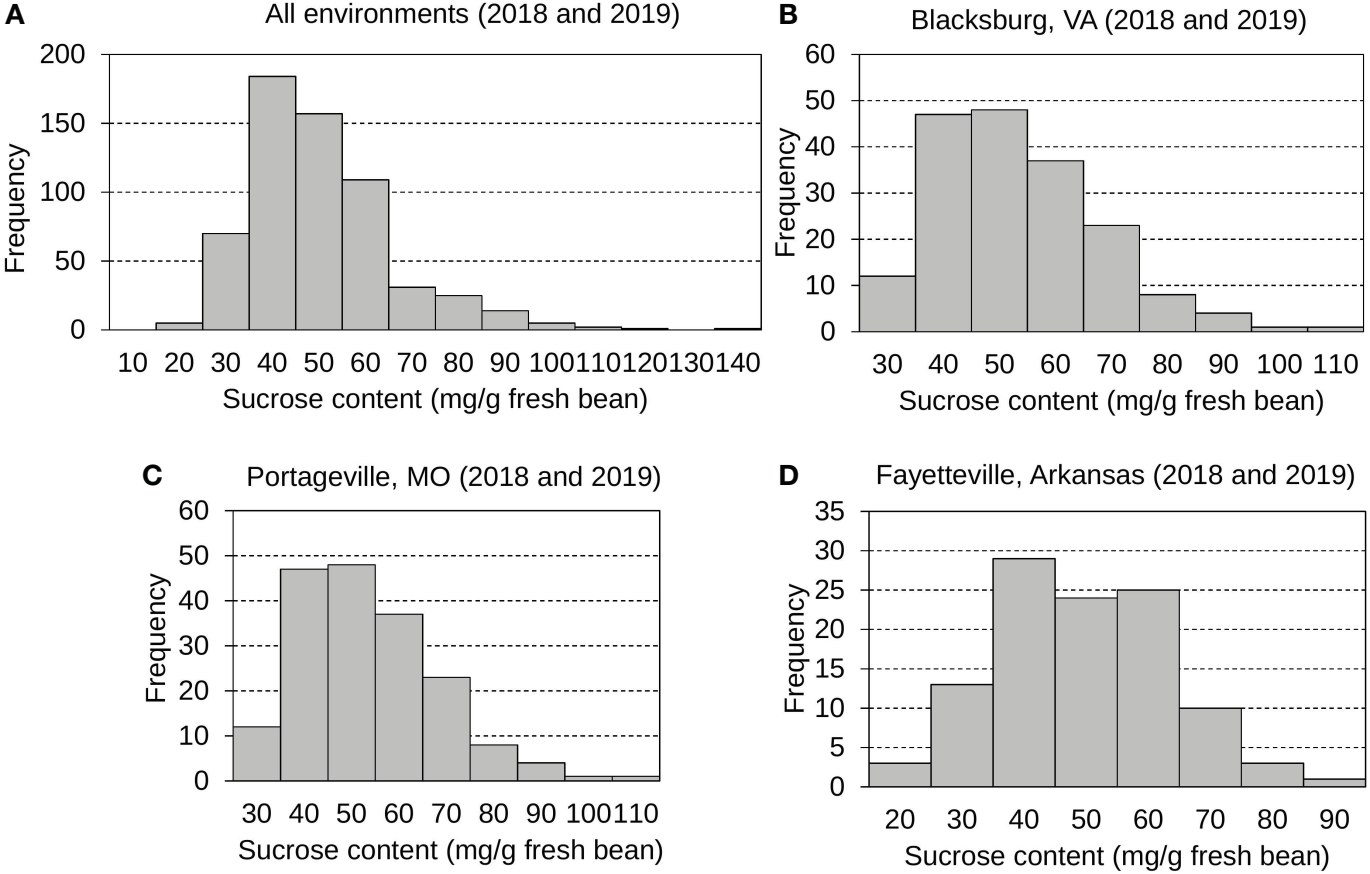
Frequency distributions displaying edamame bean sucrose contents collected from all environments **(A)**, Blacksburg, VA **(B)**, Portageville, MO **(C)**, and Fayetteville, AR **(D)**.

The mean, standard deviation, and distribution of sucrose content were calculated and analyzed (**Figure 1** and **Table S2**). Sucrose contents across all environments displayed normal, continuous distributions with a grand mean of 47.84 mg g ^-1^ fresh bean and an average standard deviation (SD) of 16.06 mg g ^-1^ fresh bean. Edamame sucrose distributions were displayed for all environments (**Figure 1A**), Blacksburg 2018 and 2019 (**Figure 1B**), Portageville 2018 and 2019 (**Figure 1C**), and Fayetteville 2018 and 2019 (**Figure 1D**). Specifically, Blacksburg 2018, 2019; Portageville 2018, 2019; and Fayetteville 2018, 2019 environments had means and SDs of 42.29, 65.77, 39.98, 44.47, 37.38, and 48.10 mg g ^-1^ fresh bean and 8.39, 18.34, 8.29, 14.07, 15.17, and 11.98 mg g ^-1^ g fresh bean, respectively.

**Figure 2:**
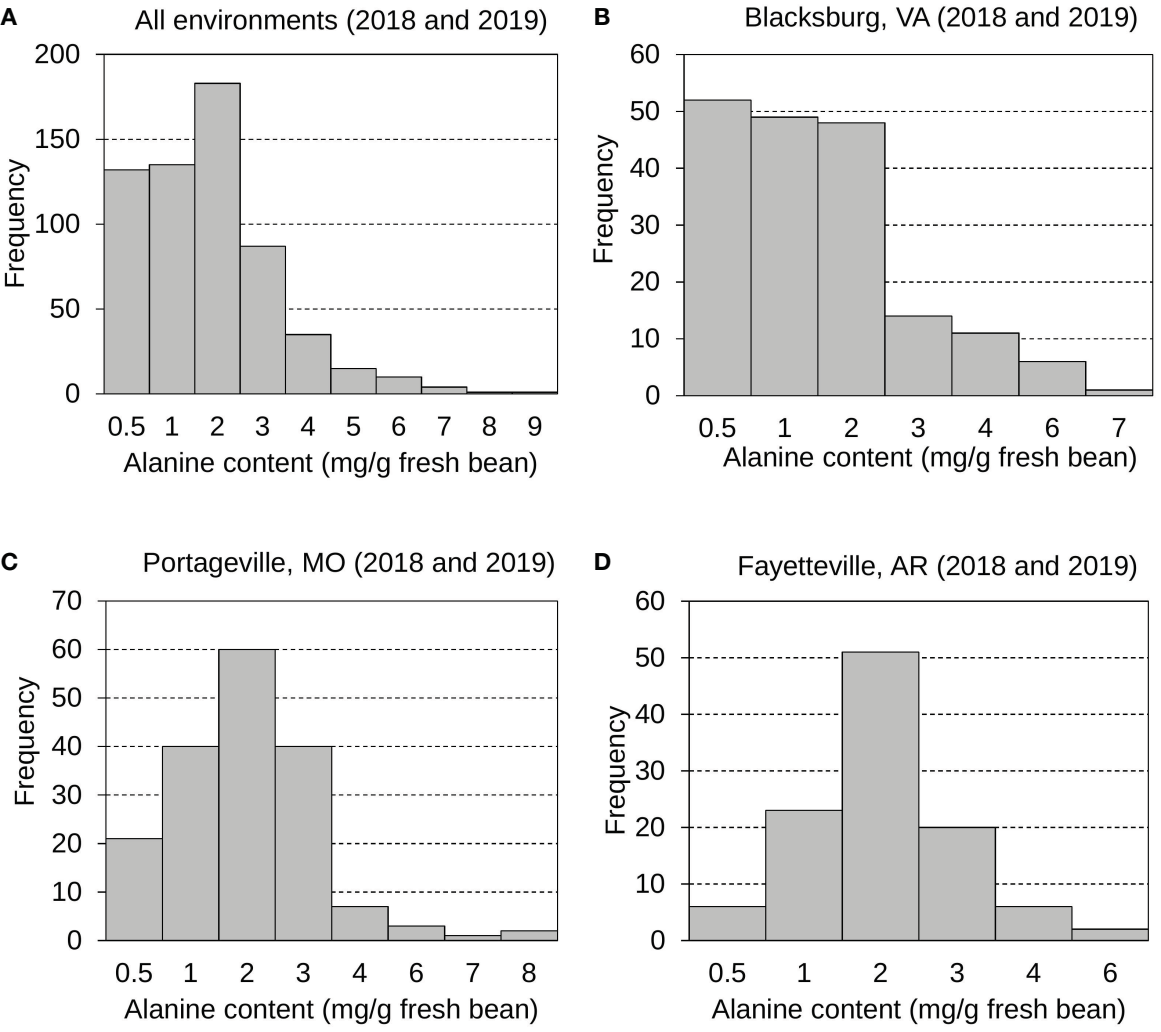
Frequency distributions displaying edamame bean proteinogenic Ala contents collected from all environments **(A)**, Blacksburg, VA **(B)**, Portageville, MO **(C)**, and Fayetteville, AR **(D)**.

As another significant contributor to edamame sweetness for customers (Yu et al., 2021), the mean, standard deviation, and distribution of Ala content in fresh edamame beans were also examined and investigated (**Figure 2** and **Table S2**). Generally, the Ala content varies more greatly than the sucrose content. Edamame Ala distributions were displayed for all environments (**Figure 2A**), Blacksburg 2018 and 2019 (**Figure 2B**), Portageville 2018 and 2019 (**Figure 2C**), and Fayetteville 2018 and 2019 (**Figure 2D**). Ala contents for Fayetteville displayed a normal, continuous distribution while it across other environments displayed skewed distributions. It had a grand mean of 1.51 mg g ^-1^ fresh bean and an average SD of 1.20 mg g ^-1^ fresh bean. Blacksburg 2018, 2019; Portageville 2018, 2019; and Fayetteville 2018, 2019 environments had Ala content means and SDs of 0.91, 1.62, 1.83, 1.60, 1.53, and 1.72 mg g ^-1^ fresh bean and 0.68, 1.54, 0.96, 1.38, 0.96, and 1.07 mg g ^-1^ g fresh bean, respectively. Samples grown in Blacksburg 2019 had significantly higher Ala content than that in 2018 while the Ala content in edamame beans from other two locations did not show notable difference across years of 2018 and 2019.

### Analysis of population structure

Although the consumption of edamame has increased in the United States in the past 20 years, most edamame is still imported from Asia (Wolfe et al., 2018). Thus, in present study, all accessions are original from East Asia, including South Korea, North Korea, Japan, mainland China, and Taiwan, China except for PI 548624, which was from Delaware, United States.

**Figure 3:**
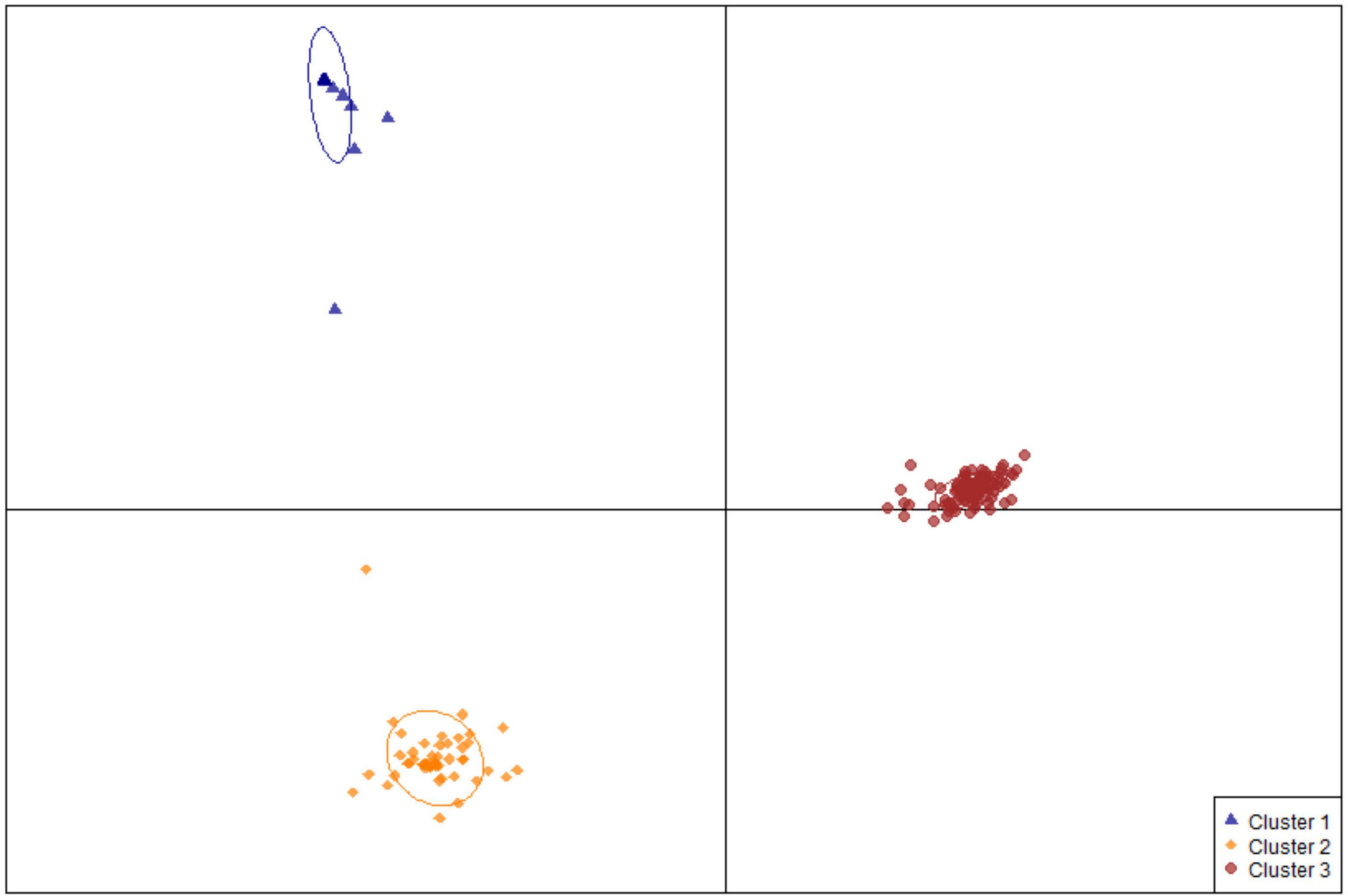
Population structure of edamame germplasms used in this study. A scatter plot depicting the three clusters identified as likely subpopulations within the 189 accessions: cluster I (blue triangle, n = 27), cluster II (gold diamonds, n = 54), cluster III (red circles, n = 107).

Using the R package DAPC, commonly used in GWAS (Jombart et al., 2010), 189 edamame accessions were classified into three genetic clusters (**Figure 3**). Cluster I has 27 accessions that are all from South Korea classified at mature group IV (**Table S1**). Cluster II has 54 accessions including 49 from South Korea, two from North Korea, two from northern China and one from Japan. With the maturity group information from ars-grin.org, the majority of the germplasm in this cluster still belongs to group IV (**Table S1**). The remaining edamame accessions were classified into cluster III with relatively high diversity in both geographic origin and maturity (**Table S1**). Specifically, this cluster includes 75 from Japan, 21 from South Korea, two from North Korea, one from the United States, 6 from mainland China, and three from Taiwan, China with maturity groups from III to VI.

### Single nucleotide polymorphisms associated with sucrose and Ala contents by GWAS

**Figure 4:**
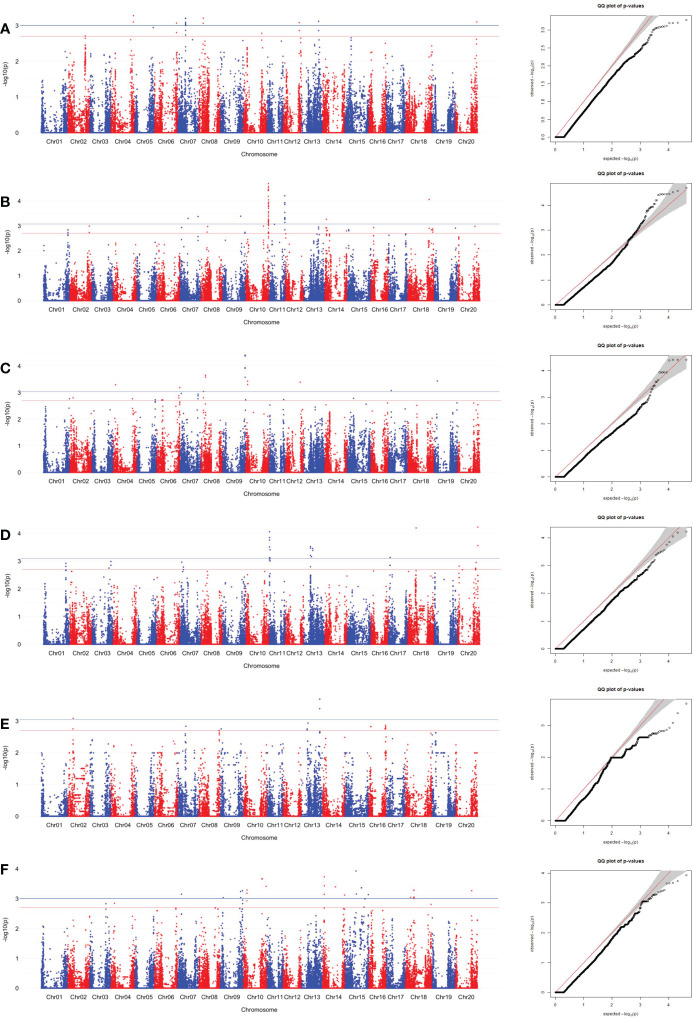
SNP associations for sucrose content in edamame beans harvest from **(A)** Blacksburg, VA, 2018, **(B)** Blacksburg, VA, 2019, **(C)** Portageville, MO, 2018, **(D)** Portageville, MO, 2019, **(E)** Fayetteville, AR, 2018, **(F)** Fayetteville, AR, 2019, are displayed in Manhattan plots with chromosomes in alternating colors, significance thresholds-log10(P) > 3.01 (the blue line) and suggestive threshold-log10(P) > 2.70 (the red line). Each respective QQ plot displays observed-log10(P) against expected-log10(P).

**Figure 5:**
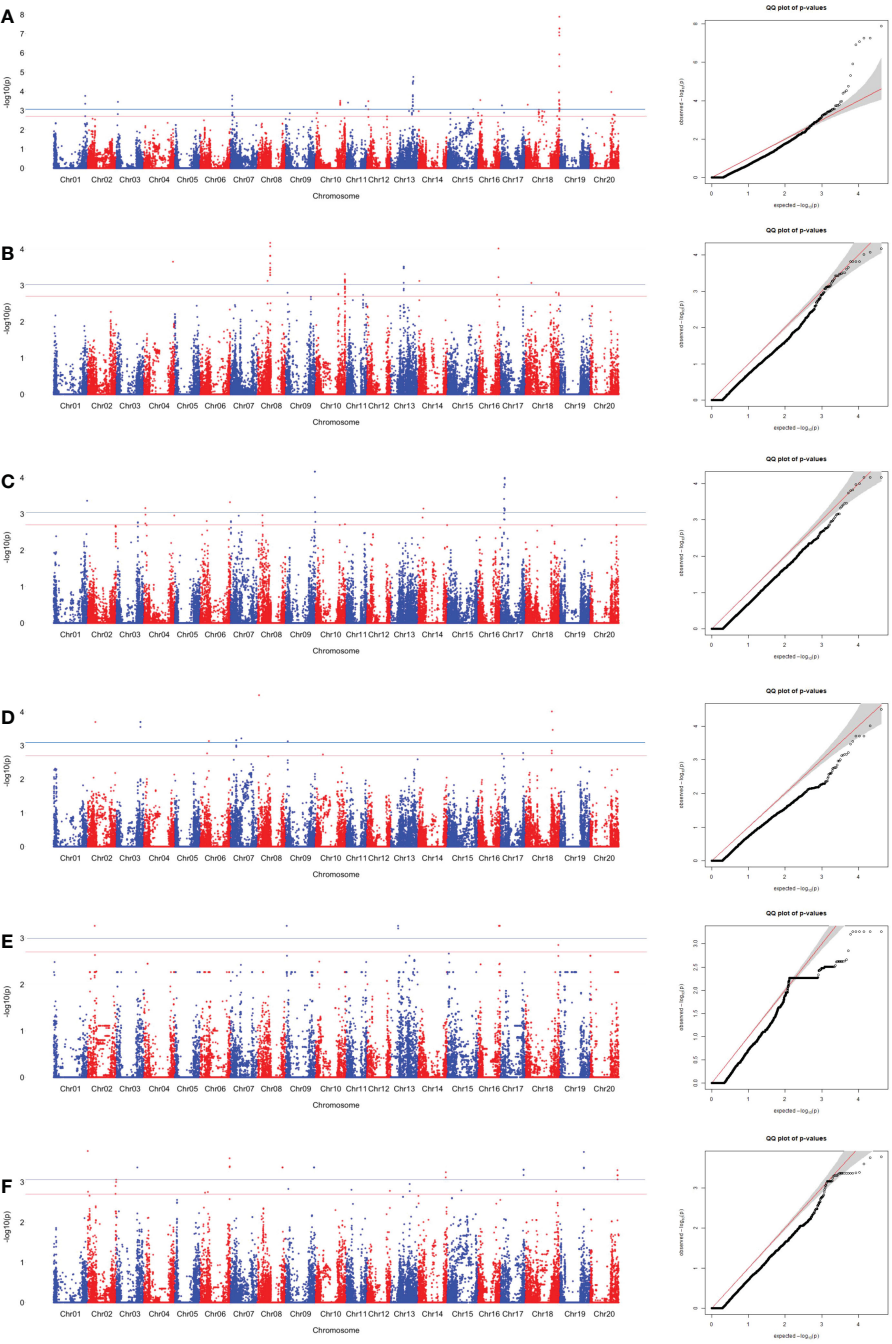
SNP associations for Ala content in edamame beans harvest from **(A)** Blacksburg, VA, 2018, **(B)** Blacksburg, VA, 2019, **(C)** Portageville, MO, 2018, **(D)** Portageville, MO, 2019, **(E)** Fayetteville, AR, 2018, **(F)** Fayetteville, AR, 2019, are displayed in Manhattan plots with chromosomes in alternating colors, significance thresholds-log10(P) > 3.01 (the blue line) and suggestive threshold-log10(P) > 2.70 (the red line). Each respective QQ plot displays observed-log10(P) against expected-log10(P).

SNPs associated with sucrose content were identified using the MLM. The SNPs associated with edamame sucrose and Ala contents were determined by significant and suggestive thresholds of -log_10_(P) > 3.01 (*α* = 0.05, the blue line in Manhattan plot in **Figures 4**, **5**, **S1**, **S2**) and -log_10_(P) > 2.70 (*α* = 0.10, the red line in Manhattan plot in **Figures 4**, **5**, **S1**, **S2**), using a modified Šidák (Li and Ji, 2005) adjustment.

A total of 43 SNPs was identified as being associated with sucrose contents in at least two phenotypic datasets (**Table S3**). **Figure 3** and **Figure S1** visualized the associations between the SNPs and sucrose content in the individual dataset and combined datasets, respectively.

Fayetteville 2018 dataset, Fayetteville 2019 dataset, and Fayetteville combined dataset had four, 16, and 22 overlapped associated SNPs, respectively; Portageville 2018 dataset, Portageville 2019 dataset, and Portageville combined dataset had three, eight, and 13 overlapped associated SNPs, respectively; and Blacksburg 2018 dataset, Blacksburg 2019 dataset, and Blacksburg combined dataset had two, five, and seven overlapped associated SNPs, respectively. In addition, BLUE combined dataset had nine overlapped associated SNPs. It was noticed that 40 SNPs associated with two phenotypic datasets while 3 SNPs associated with three phenotypic datasets. They were: ss715599697 (Chr 8) being associated with Blacksburg 2019, Portageville combined, and BLUE combined; ss715602625 (Chr 8) being associated with Portageville 2018, Fayetteville combined, and BLUE combined; ss715618513 being associated with Blacksburg combined, Fayetteville 2018 and 2019. The SNP associations are distributed on 11 chromosomes in the soybean genome: Chromosome 3, 6, 8, 9, 10, 13, 14, 16, 17, 18, and 20. Chr 18 contained 12 SNP associations, which was the most, followed by seven SNP associations in Chr 13, five SNP associations in both Chr 14 and Chr 8, three SNP associations in both Chr 6 and Chr 20, two SNP associations in three chromosomes including Chr 9, Chr 16 and Chr 17, one SNP association in both Chr 3 and Chr 10.

A total of 25 SNPs was identified as being associated with Ala contents in edamame beans in at least two phenotypic datasets (**Table S4**). **Figure 5** and **Figure S2** visualized the associations between the SNPs with the sucrose content in the individual dataset, and combined datasets, respectively. BLUE combined dataset had the 19 overlapped associated SNPs, what was the most among all the phenotypic datasets. Fayetteville 2018 dataset, Fayetteville 2019 dataset, and Fayetteville combined dataset had one, three, and four overlapped associated SNPs, respectively; Portageville 2018 dataset, Portageville 2019 dataset, and Portageville combined dataset had five, one, and eight overlapped associated SNPs, respectively; and Blacksburg 2018 dataset and Blacksburg combined dataset had seven and six overlapped associated SNPs, respectively.

Although there were 35 (above the significant threshold) and 20 (above the suggestive threshold) SNPs being identified to associate with Alaine content in the Blacksburg 2019 dataset (**Figure 4**), none of them overlapped with the SNPs derived by other phenotypic datasets. Twenty-one SNPs associated with two phenotypic datasets while four SNPs associated with three phenotypic datasets. Interestingly, the four SNPs were adjacent in the Chr 8: ss715628063, ss715628063, ss715628067 all being associated with Portageville 2018, Portageville combined, and BLUE combined while ss715628064 being associated with Blacksburg 2018, Portageville 2018, and BLUE combined. The associations distributed in nine chromosomes in soybean genome: Chr 2, 6, 8, 10, 11, 13, 14, 17, 18. Chr 17 contained the 10 SNP associations, which was the most, followed by five SNP associations in Chr 14, four SNP associations in Chr 13, and one SNP association in Chr 2, Chr6, Chr 8, Chr 10, Chr 11, and Chr 18.

### Prediction of candidate genes controlling sucrose content

In total, 39 sucrose-related candidate genes from WM82 were found within 10 kb flanking regions of individual significant SNP (**Table S5**). A number of gene models were found on eleven chromosomes: one on Chr 3, three on Chr 6, four on Chr 8, two on Chr 9, one on Chr 10, seven on Chr 13, five on Chr 14, two on Chr 16, two on Chr 17, nine on Chr 18, three on Chr 20 (**Table S5**). These candidate genes have various functions in the numerous biological and biochemical processes. Interestingly, there were two genes (Glyma.08g137500 and Glyma.10g270800) being annotated to function as the trehalose-6-phosphate synthase (TPS). TPS is able to catalyze the production of trehalose-6-phosphate (T6P), which can convert to trehalose after consecutive dephosphorylation by trehalose-6-phosphate phosphatase (TPP) (Ponnu et al., 2011; Yadav et al., 2014). T6P has been shown to regulate sucrose utilization in plants since the precursors of T6P are derived from the sucrose metabolism (Yadav et al., 2014). Thus, the two genes might serve as a key component regulating the sucrose content in edamame beans.

In addition, there were two SNPs (ss715607940 and ss715631133) being identified only associated with the sucrose content in the edamame beans harvested by Blacksburg 2019. Within the flanking regions of these two significant SNPs, two genes were found to have the potential to control the sucrose content. Glyma.18g193600 and Glyma.10g268500 located near the ss715631133 and ss715607940 on Chr 18 and Chr 10, respectively. Glyma.18g193600 encoded a Fructose-1,6-bisphosphatase (FBPase, EC:3.1.3.11) protein, which was characterized as a key enzyme in the plant sucrose synthesis pathway, in the Calvin cycle (Ge et al., 2020). Glyma.10g268500 encoded a Fructose-1, 6-bisphosphate aldolase (FBA, EC 4.1.2.13) protein enzyme that is a key plant enzyme being involved in glycolysis, gluconeogenesis, and the Calvin cycle (Lv et al., 2017). It is worthy to investigate the potential function of the fructose-metabolism-related genes on regulating the edamame bean sucrose content for the basic edamame genetics research and applied edamame breeding.

### Prediction of candidate genes controlling Ala content

In total, 25 Ala-related candidate genes from WM82 were found within 10 kb flanking regions of each significant SNP (**Table S6**). A number of gene models were found on nine chromosomes: one on Chr 2, one on Chr 6, one on Chr 8, one on Chr 10, one on Chr 11, four on Chr 13, five on Chr 14, 10 on Chr 17, and one on Chr 18 (**Table S6**). Based on the annotated information in the soybase, these candidate gene models belong to several protein families with various metabolic and biosynthesis implications including DNA binding, development and programmed cell death, protein kinase, etc. It is noteworthy there were two genes (Glyma.14g201100 andGlyma.18g269600) being annotated to function as the O-methyltransferase (OMT) while the gene of Glyma.17g070500 was annotated to encode a S-adenosylmethionine (SAM) decarboxylase. Both OMT and SAM are involved in the biochemical pathway from methionine to cysteine, which is located at the upstream pathway from cysteine to Ala (Morishige et al., 2010; Hildebrandt et al., 2015). It implies that the three candidate genes above might have the potential in controlling metabolism of Ala in edamame beans that is worthy for further validation and investigation.

## Discussion

The sucrose and Ala in edamame beans are two desirable traits for sweet taste and flavor of edamame and its derived food product to promote edamame as a nutritional specialty crop in the world. Therefore, varieties with high sucrose and Ala levels are urgently needed in the edamame industry. In the current study, 189 potential edamame accessions with large seed size (> 20 g/100 seeds) (**Table S1**) were collected. The sucrose and Ala contents were determined in the seeds obtained from three states for two years. A GWAS study was employed to explore the genetic controls for these two traits using SoySNP50K iSelect BeadChip (Song et al., 2013).

### Fluctuation of sucrose and Ala levels in different environments

Major seed composition characteristics, such as amino acid, oil, and sucrose content, are significantly affected by environments (Bandillo et al., 2015; Chaudhary et al., 2015). The sucrose and Ala levels in the edamame beans were different across three locations (**Figures 1**, **2** and **Table S2**). It might be due to the diversity of temperatures and precipitation rates among three locations, affecting plant growth and/or seed development (Zeng et al., 2014). It was reported that decreased soil moisture content increased the sucrose level (Wang et al., 2021). Blacksburg (VA), 2019 had a much higher precipitation (42.15 inches) than 2018 (59.35 inches), which may explain why the average sucrose content in edamame harvested by Blacksburg 2019 was higher than that in 2018. The similar negative relationship was also observed between the edamame bean sucrose content and the precipitation level at the location of Portageville, MO. It had 48.57 inches precipitation in the year of 2018 and an average sucrose content was 39.98 mg g^-1^ fresh bean, while it had 61.84 inches precipitation in the year of 2019 and an average sucrose content was 44.47 mg g^-1^ fresh bean. However, the similar trend was not found between the edamame bean sucrose content and the precipitation level at the location of Fayetteville, AR. It implies that the sucrose content in fresh edamame bean is a complex trait affected by multiple environmental factors and more research efforts need to be invested to decouple their relationship. In contrast to sucrose content, Ala content was distributed more divergently. There were few reports characterizing the genetic basis of Ala synthesis and signaling in the soybean/edamame beans. Our study can lay a foundation of understanding the impact of environmental factors on the edamame Ala content in order to boost future breeding edamame varieties with sweeter flavor.

### Potential edamame breeding PIs

The long-term goal of this study is to serve as the foundation for breeding edamame varieties with improved sensory adapted to distinctive environments under the global climate change challenge. Therefore, in addition to investigating the genetic causes regulating sucrose and Ala contents in edamame beans by GWAS, there is an urgent need to select a collection of sweet edamame germplasms with moderate diversity.

By summarizing the sucrose and Ala contents in the edamame beans, three PI accessions were selected for their high sucrose and high Ala content. PI 532469, PI 243551, and PI 407748 had sucrose content means and SDs of 63.69, 71.67, 65.01 mg g^-1^ fresh bean and 6.53, 29.3, 16.91 mg g^-1^ fresh bean while they had Ala content means and SDs of 4.60, 3.73, 3.62 mg g^-1^ fresh bean and 1.42, 2.75, 1.90 mg g^-1^ fresh bean. They had high content of sucrose and Ala in more than one tested environment, displaying consistent sweetness. The three “sweet” accessions characterized in present study had the comparable sucrose content but significantly high Ala content. By contrast, VT Sweet, a recent released edamame cultivar (Zhang et al., 2021), had 69.22 ± 21.65 mg sucrose g^-1^ fresh bean and 0.59 ± 0.07 mg Ala g^-1^ fresh bean in the Blacksburg extension trial, 2019. PI532469 and PI243551 both originated from Japan and PI407748 originated from China. PI243551 and PI407748 belong to maturity group III while the information of maturity information remains unclear for PI532469. These three accessions can serve as genetic resources in edamame breeding to provide sweet trait.

### The population structure of the edamame germplasm

As shown in **Figure 3** and **Table S1**, 188 out of 189 edamame accessions used in this study originated from East Asia whereas only PI548624 is from the United States, mainly because soybean is original from China, and the communities in East Asia started consuming edamame centuries ago. The current edamame germplasm from East Asia provides the materials for other global regions, including the United States for edamame breeding.

Although the geographical origins of 188 germplasms is East Asia, the R package DAPC was still able to distinguish them (Jombart et al., 2010). All 27 accessions in cluster I and 49 out of 54 accessions in cluster II originated from South Korea (**Table S1**). In addition, most accessions in these two clusters belong to maturity group IV (**Table S1**), implying their potential adaptability to the south-central United States. The edamame germplasms in cluster III display a higher divergency in both geographic origin and maturity (**Table S1**). They were documented originally in Japan, South Korea, North Korea, mainland China, and Taiwan, China with maturity groups of III, IV, V or even later (**Table S1**). The diverse germplasms in cluster III have the potential to promote the edamame as a specialty crop adapting to all states of America.

### Application of BLUE combined dataset to GWAS

In present study, the sucrose and Ala contents were measured in the edamame beans harvested from six environmental combinations (location × year). However, because of the environmental variation and genotype by environment interaction, the SNPs associated with sucrose and Ala were different at each environment. To diminish the variations caused by local environments and fluctuation of annual weather conditions, adjusted phenotype based on BLUE was used to characterize sucrose and Ala related genes in the edamame beans (Haslett and Puntanen, 2009). The sucrose and Ala contents of six combinations were combined for the phenotyping input. With the combined data, two SNPs coupling with Ala and one SNP associated with sucrose were identified, which were different from the SNPs derived from the phenotyping data of combinations of certain location × certain year. The functions of these SNPs in the sucrose and Ala signaling require to be validated by further experimental evidence.

### Candidate genes and signaling pathways related to edamame sucrose content

Soluble sugar content in soybean seeds, especially sucrose content, significantly affects the taste and flavor of soybean foods such as soy milk and tofu (Kim et al., 2005). In addition to its important nutritional value in seeds, sucrose is the main photosynthesis product of higher plants. It is not only a carbon base for plant physiological metabolism, but also a signal molecule that coordinates the relationship between plant sources and sinks. Sucrose is involved in various metabolic and biosynthetic processes and plays a crucial role in plant growth and seed development (Ruan, 2014). Furthermore, sucrose has important functions in responses to various abiotic stresses (Rosa et al., 2009; Du et al., 2020).

In this study, a total of 41 candidate genes were screened in genomic regions of 45 SNPs (**Table S5**). Further investigations of their biological functions can assist selection for edamame germplasms with higher sucrose content. Of the candidate genes, four leucine-rich repeat-containing genes were identified (**Table S5**). Leucine-rich repeats (LRR) containing genes represent a large and complex gene family in plants, and are functional in various biological and biochemical processes including development, plant immunity, and responses to environmental stimuli (Dufayard et al., 2017). Essential for plant growth and development, sucrose is engaged in plant defense by activating plant immune responses against pathogens (Tauzin and Giardina, 2014). During infection, pathogens reallocate the plant sugars for their own needs forcing the plants to modify their sugar content and triggering their defense responses (Tauzin and Giardina, 2014). Thus, we assume that the LRR domain contains genes allied with sucrose content that might be involved in the plant defense behavior mediated by sucrose signaling.

In addition, 18 enzyme-type genes with catalytic function and two kinase-type genes were also characterized to associate with sucrose content in the edamame beans (**Table S5**). Due to the importance of sucrose for plants, its synthesis, accumulation and signaling in plants are regulated with a subtle manner. A large number of enzymes along with kinases have been reported in charge of sucrose homeostasis. For instance, the members in two kinase families including SNRK and CDPK, are broadly demonstrated to participate in the synthesis, transportation, and signaling of sucrose (Hulsmans et al., 2016; Crizel et al., 2020). The functions of candidate enzyme and kinase genes derived from GWAS analysis of sucrose content in this study will be noteworthy to investigate to reveal the sucrose related mechanism in soybean seeds and edamame beans.

To date, 37 QTL and 362 genes were identified that are related with sucrose content in dried soybean seeds (https://www.soybase.org/search/index.php?searchterm=sucrose). By comparison with these reported genetic sources responsible for soybean sucrose content, two genes (Gm10g268500 and Gm18g193600) obtained from this study were confirmed to have effects in regulating sucrose content and encode two enzyme proteins that locate adjacently in the sucrose synthesis pathway. The first one is FBA, encoded by Gm10g268500. It catalyzes the reversible aldol cleavage of fructose-1,6-bisphosphate (FBP) into dihydroxyacetone phosphate (DHAP) and glyceraldehyde-3-phosphate (GAP), either in glycolysis or gluconeogenesis and in the Calvin-Benson cycle (Lv et al., 2017). The second gene is FBPase, encoded by Gm18g193600. It catalyzes the conversion of FBP to fructose-6-phosphate in the Calvin cycle and eventually to starch in chloroplast, while it catalyzes the conversion of triose phosphates to sucrose in the sucrose-biosynthesis pathway in cytosol (Serrato et al., 2009). Both FBA and FBPase are the key enzymes in the plant sucrose synthesis pathway, in the Calvin cycle, and also play important roles in photosynthesis regulation in green plants (Serrato et al., 2009; Lv et al., 2017). These reactions are essential for carbon fixation and sucrose metabolism. The results confirmed once again that the two genes have solid effects in regulating sucrose content. Hence, the value of these two genes for increasing the content of sucrose in edamame was worth exploring in the future.

In addition to FBPase and FBA genes, two TPS genes, Gm10g270800 and Glyma.08g137500), were unveiled to couple with sucrose content in the edamame beans. TPS catalyzes the synthesis of T6P from UDP-glucose (UDPG) and glucose-6-P (G6P), which are derived from the sucrose metabolism (Ponnu et al., 2011; Yadav et al., 2014). A regulatory loop consisting of T6P, SnRK1, and bZIP11 is reported controlling sucrose availability and utilization (Delatte et al., 2011). In addition, T6P has recently emerged as an important signaling metabolite, regulating carbon assimilation, plant sugar status, and plant development (Ponnu et al., 2011; Zhang et al., 2017). Our results suggested that TPS and T6P-related metabolism might be involved in manipulating edamame bean sucrose content, which has never been reported in grain type soybean basic genetics. It implies the genetic base for sucrose content in fresh edamame bean might vary from that in dried soybean seed and provide a novel insight for edamame bean sucrose genetics as well as a promising foundation for edamame breeding with improved flavor.

### Candidate genes and signaling pathways related to edamame Ala content

Catabolic pathways for Ala in plants is very short, where cysteine desulfurase (EC 2.8.1.7) catalyzes the conversion from cysteine to Ala, and Ala is directly converted to pyruvate by Ala aminotransferase (EC 2.6.1.2) (Hildebrandt et al., 2015). Labeling experiments using ^13^CO_2_ revealed that *de novo* biosynthesis rate of Ala was much rapidly than several other amino acids including phenylAla, glycine, and serine, glutamate, threonine, lysine, leucine, isoleucine, proline, and asparagine (Hildebrandt et al., 2015). This result is consistent with the relatively simple and short synthesis pathway of Ala. To date, only 30 candidate genes and six QTL were recorded to be associated with Ala content in soybean plants (https://www.soybase.org/search/index.php?searchterm=Ala). The quantity of genetic sources impacting the Ala content is much less than the essential amino acids. Compared with grain-type soybean, Ala content in edamame beans is extremely important because it is one of the two major contributors for the sweetness of edamame bean and partially determines consumer acceptance. Additionally, Ala was reported as a generic stress response molecule involved in protecting plants from extreme temperature, hypoxia, drought, and heavy metal shock (Parthasarathy et al., 2019). There was also evidence that Ala participated in lignin biosynthesis and ethylene production. The present study can assist the genetic understanding of Ala metabolism in the soybean/edamame beans and guide the edamame breeding with a goal of more sweetness.

A total of 25 candidate genes were screened in the respective genomic regions of 25 SNPs (**Table S6**). In the future, the SNPs and/or loci with validated function will be capable to boost selection for edamame with higher Ala content germplasms. Three genes encoding DNA-binding proteins and two kinase genes were demonstrated to be associated with Ala content (**Table S6**). None of the above genes was strongly overlapped with the current known Ala-allied QTL and genes obtained from the soybase, probably because they are genes responsible for Ala content in edamame beans other than the dried soybean seeds. Their functions would be explored in the future with the intention of uncovering the mechanism of Ala synthesis, accumulation, transportation and signaling.

Additionally, nine enzyme-type genes with catalytic functions were found to associate with edamame Ala content (**Table S6**). Uniquely, a guanosine monophosphate (GMP) synthetase (EC 6.3.5.2) gene was revealed to associate with Ala content in the edamame beans (**Table S6**). GMP synthetase is an amidotransferase that catalyzes the amination of xanthosine 5’-monophosphate to form GMP in the presence of glutamine and ATP. Glutamine hydrolysis produces the necessary amino group while ATP hydrolysis drives the reaction (Nakamura et al., 1995). Moreover, GMP synthetase is involved in amino acid metabolism because it generates L-glutamate from L-glutamine (Yoo et al., 2020). Hence, we suspected that GMP synthetase genes might be also involved in the Ala generation and homeostasis in edamame beans.

A SAM decarboxylase gene (Gm17g070500) was identified to ally with Ala content (**Table S6**). SAM is a key intermediate component linking the conversion from methionine and cysteine, which locates at the upstream of the Ala synthesis pathway (Hildebrandt et al., 2015). Additionally, two OMT genes (Glyma.14g201100 and Glyma.18g269600) were found to link with Ala content. OMT is a type of methyltransferase enzyme transferring a methyl group on a molecule (Morishige et al., 2010). Similarly, OMT was reported being engaged in the biochemical pathway from methionine to cysteine, followed by the Ala synthesis pathway. Nevertheless, the direct evidence for the impact of these gene on the Ala signaling pathway and synthesis remains unclear yet. In the future, we will employ *in vitro* enzyme assay trial to explore its substrate and biochemical function, and might apply the gene editing tool to characterize this gene’s *in vivo* function related to Ala.

## Conclusion

By analysis of sucrose and Ala levels in 189 edamame accessions at three locations in two years, three PI accessions were selected as the high sucrose and high Ala accessions that are able to serve the breeding of sweeter edamame varieties. A total of 45 and 25 SNPs was identified to associate sucrose content and Ala content, respectively, based on phenotypic data derived 189 edamame accessions and SoySNP50K iSelect BeadChip by GWAS. The PI accessions, beneficial alleles, and candidate genes identified in this study will be fundamental for breeding sweeter edamame varieties with improved seed levels of sucrose and Ala, and eventually promote the consumers’ acceptance of edamame as a specialty crop in the United States.

## Data availability statement

The original contributions presented in the study are included in the article/**Supplementary Material**. Further inquiries can be directed to the corresponding author.

## Author contributions

ZW and BZ designed the experiment. BZ selected the edamame panel and provided plant samples. BZ, NL, LM, PC led the field trials at VA, MO and AR. DY led the measurements of sucrose and Ala contents in the edamame beans. KD and GM combined the phenotypic data using BLUE. ZW, KD, GM and SL did the GWAS analysis. ZW, DY, and GM wrote the manuscript. All authors contributed to the article and approved the submitted version.

## Supplementary material

Supplementary Figure 1 SNP associations for edamame sucrose content from **(A)** BLUE combined dataset, **(B)** Blacksburg, VA, combined, **(C)** Portageville, MO, combined, **(D)** Fayetteville, AR, combined, are displayed in Manhattan plots with chromosomes in alternating colors, significance thresholds-log10(P) > 3.01 (the blue line) and suggestive threshold-log10(P) > 2.70 (the red line). Each respective QQ plot displays observed-log10(P) against expected-log10(P).

Supplementary Figure 2 SNP associations for edamame Ala content from **(A)** BLUE combined dataset, **(B)** Blacksburg, VA, combined, **(C)** Portageville, MO, combined, **(D)** Fayetteville, AR, combined, are displayed in Manhattan plots with chromosomes in alternating colors, significance thresholds-log10(P) > 3.01 (the blue line) and suggestive threshold-log10(P) > 2.70 (the red line). Each respective QQ plot displays observed-log10(P) against expected-log10(P).

Supplementary Table 1 Germplasms utilized in present study and their populational structure

Supplementary Table 2 Sucrose and Ala contents in the edamame beans harvested from Blacksburg (VA), Portageville (MO), and Fayetteville (AR) in the years of 2018 and 2019.
