# Supplementary figures and images for: Genome-wide association analysis of sucrose and alanine contents in edamame beans
Source: Front Plant Sci. 2023 Feb 3;13:1086007. doi: 10.3389/fpls.2022.1086007 (PMC9935843; doi:10.3389/fpls.2022.1086007)

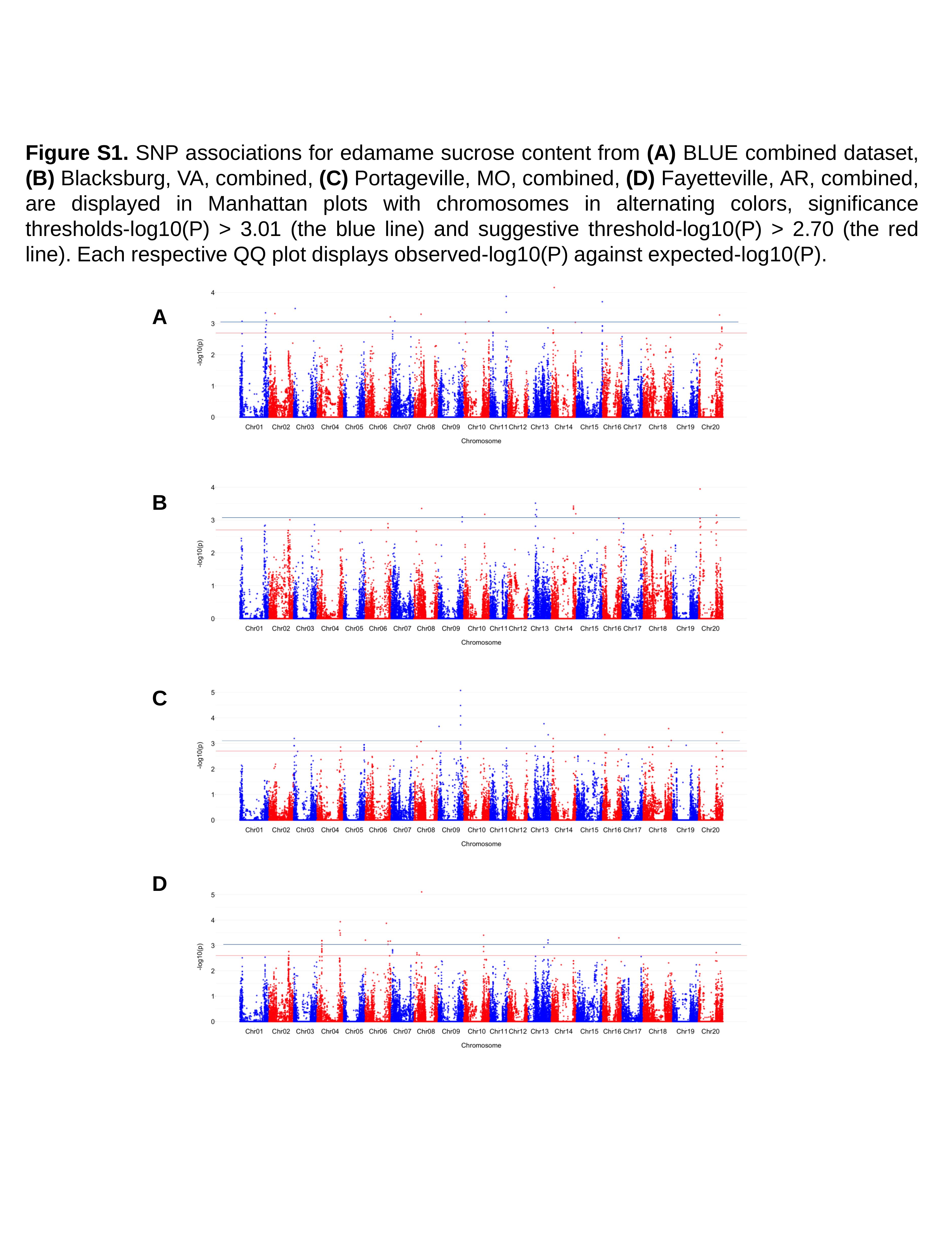

Supplement: Supplementary Figure 1 — SNP associations for edamame sucrose content from (A) BLUE combined dataset, (B) Blacksburg, VA, combined, (C) Portageville, MO, combined, (D) Fayetteville, AR, combined, are displayed in Manhattan plots with chromosomes in alternating colors, significance thresholds-log10(P) > 3.01 (the blue line) and suggestive threshold-log10(P) > 2.70 (the red line). Each respective QQ plot displays observed-log10(P) against expected-log10(P). [file Image_1.jpeg]

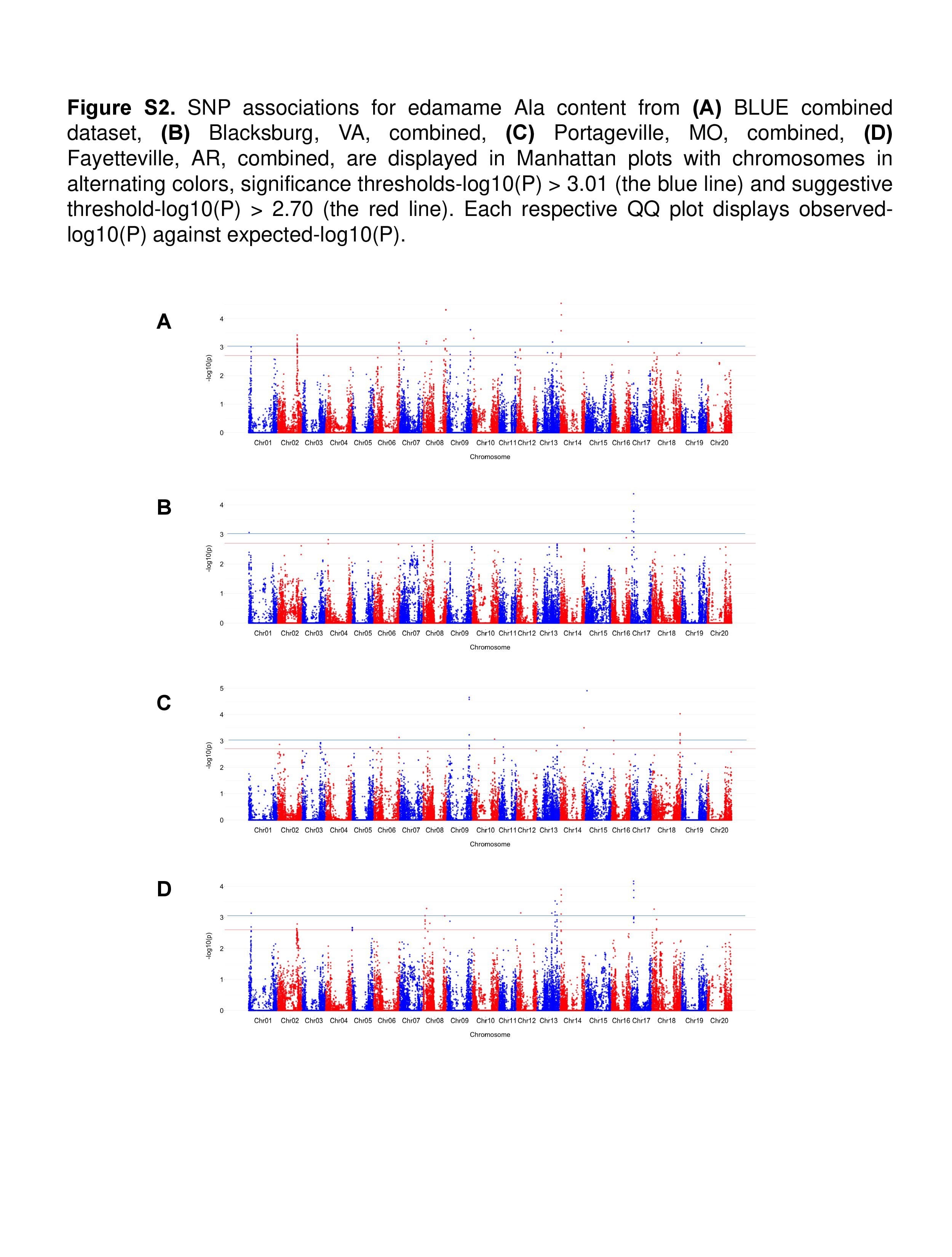

Supplement: Supplementary Figure 2 — SNP associations for edamame Ala content from (A) BLUE combined dataset, (B) Blacksburg, VA, combined, (C) Portageville, MO, combined, (D) Fayetteville, AR, combined, are displayed in Manhattan plots with chromosomes in alternating colors, significance thresholds-log10(P) > 3.01 (the blue line) and suggestive threshold-log10(P) > 2.70 (the red line). Each respective QQ plot displays observed-log10(P) against expected-log10(P). [file Image_2.jpeg]
