# Supplementary material for: Genome-wide association analysis of sucrose and alanine contents in edamame beans
Source: Front Plant Sci. 2023 Feb 3;13:1086007. doi: 10.3389/fpls.2022.1086007 (PMC9935843; doi:10.3389/fpls.2022.1086007)
Supplement: Supplementary file 5 [file Table_3.docx]

**Table S3.** Significant SNPs associated with the edamame sucrose content

| Chromosome | SNP | WM82^a^ Allele | Alternative Allele | Environments^b^ | | | | | | | | | |
| --- | --- | --- | --- | --- | --- | --- | --- | --- | --- | --- | --- | --- | --- |
|  |  |  |  | Blacksburg, VA | | | Portageville, MO | | | Fayetteville, AR | | | BLUE |
|  |  |  |  | 2018 | 2019 | combined | 2018 | 2019 | combined | 2018 | 2019 | combined |  |
| Chr03 | loc_ss715586179 | C | T | NS | NS | NS | NS | 0.001413* | 0.001380* | NS | NS | NS | NS |
| Chr06 | loc_ss715594500 | C | T | NS | NS | NS | NS | NS | 0.001697* | NS | NS | NS | 0.000908** |
|  | loc_ss715594504 | T | C | NS | NS | NS | NS | NS | 0.001720* | NS | NS | NS | 0.000687** |
|  | loc_ss715595091 | G | A | NS | NS | 0.000611** | NS | NS | NS | NS | NS | NS | 0.000676** |
| Chr08 | loc_ss715599195 | C | T | 0.000621** | NS | NS | 0.000224** | NS | NS | NS | NS | NS | NS |
|  | loc_ss715599209 | C | A | 0.000863** | NS | NS | 0.000261** | NS | NS | NS | NS | NS | NS |
|  | loc_ss715599697 | G | A | NS | 0.001060* | NS | NS | NS | 0.000444** | NS | NS | NS | 0.000008** |
|  | loc_ss715602625 | T | C | NS | NS | NS | 0.001554* | NS | NS | NS | NS | 0.001306* | 0.001951* |
| Chr09 | loc_ss715603160 | A | C | NS | NS | NS | NS | NS | NS | 0.001779** | NS | 0.000217** | NS |
|  | loc_ss715604467 | G | A | NS | NS | NS | NS | NS | NS | NS | 0.000577** | 0.000008** | NS |
| Chr10 | loc_ss715607940 | G | A | NS | 6.59E-05 | NS | NS | NS | NS | NS | NS | NS | NS |
|  | loc_ss715607973 | G | A | NS | 0.000037* | 0.000840** | NS | NS | NS | NS | NS | NS | NS |
| Chr13 | loc_ss715615127 | T | C | NS | NS | NS | NS | NS | NS | NS | NS | 0.000170** | 0.001175* |
|  | loc_ss715616068 | C | T | NS | NS | 0.001364* | NS | NS | NS | NS | NS | NS | 0.000795** |
|  | loc_ss715616116 | C | T | NS | NS | NS | NS | NS | NS | 0.000205** | NS | 0.000461** | NS |
|  | loc_ss715616737 | C | T | NS | NS | NS | NS | 0.000621** | 0.000779** | NS | NS | NS | NS |
|  | loc_ss715617105 | A | G | NS | NS | NS | NS | 0.000299** | 0.000686** | NS | NS | NS | NS |
|  | loc_ss715617106 | C | A | NS | NS | NS | NS | 0.000341** | 0.000306** | NS | NS | NS | NS |
|  | loc_ss715617107 | G | A | NS | NS | NS | NS | 0.000621** | 0.001543* | NS | NS | NS | NS |
|  | loc_ss715617181 | A | G | NS | NS | NS | NS | NS | NS | 0.001169* | NS | 0.001304* | NS |
| Chr14 | loc_ss715618513 | G | A | NS | NS | 0.001948* | NS | NS | NS | NS | 0.000186** | 0.000646** | NS |
|  | loc_ss715618537 | A | C | NS | NS | NS | NS | NS | NS | NS | 0.000371** | 0.001321* | NS |
|  | loc_ss715618543 | T | C | NS | 0.001235* | 0.001605* | NS | NS | NS | NS | NS | NS | NS |
|  | loc_ss715619362 | A | G | NS | 0.001606* | 0.000904** | NS | NS | NS | NS | NS | NS | NS |
|  | loc_ss715619687 | T | C | NS^c^ | 0.001520* | 0.000069** | NS | NS | NS | NS | NS | NS | NS |
| Chr16 | loc_ss715624465 | T | G | NS | NS | NS | NS | NS | 0.000878** | NS | NS | NS | 0.000503** |
|  | loc_ss715625088 | T | C | NS | NS | NS | NS | NS | NS | 0.001510* | NS | 0.000456** | NS |
| Chr17 | loc_ss715626713 | G | A | NS | NS | NS | NS | 0.001423* | 0.001283* | NS | NS | NS | NS |
|  | loc_ss715626717 | T | G | NS | NS | NS | NS | 0.001423* | 0.001858* | NS | NS | NS | NS |
| Chr18 | loc_ss715629456 | G | A | NS | NS | NS | NS | NS | NS | NS | 0.000897** | 0.001409* | NS |
|  | loc_ss715629459 | C | T | NS | NS | NS | NS | NS | NS | NS | 0.000897** | 0.001409* | NS |
|  | loc_ss715629461 | G | T | NS | NS | NS | NS | NS | NS | NS | 0.000897** | 0.001409* | NS |
|  | loc_ss715629462 | T | C | NS | NS | NS | NS | NS | NS | NS | 0.000897** | 0.001409* | NS |
|  | loc_ss715629463 | G | A | NS | NS | NS | NS | NS | NS | NS | 0.000897** | 0.001409* | NS |
|  | loc_ss715629466 | G | A | NS | NS | NS | NS | NS | NS | NS | 0.000897** | 0.001409* | NS |
|  | loc_ss715629470 | C | T | NS | NS | NS | NS | NS | NS | NS | 0.000897** | 0.001409* | NS |
|  | loc_ss715629479 | T | C | NS | NS | NS | NS | NS | NS | NS | 0.000897** | 0.001409* | NS |
|  | loc_ss715629481 | C | T | NS | NS | NS | NS | NS | NS | NS | 0.000897** | 0.001409* | NS |
|  | loc_ss715629483 | G | A | NS | NS | NS | NS | NS | NS | NS | 0.000897** | 0.001409* | NS |
|  | loc_ss715629508 | C | A | NS | NS | NS | NS | NS | NS | NS | 0.000897** | 0.001409* | NS |
|  | loc_ss715629516 | C | T | NS | NS | NS | NS | NS | NS | NS | 0.000897** | 0.001409* | NS |
|  | loc_ss715631133 | G | A | NS | 8.80E-05 | NS | NS | NS | NS | NS | NS | NS | NS |
| Chr20 | loc_ss715637331 | C | A | NS | NS | NS | NS | 0.001516* | 0.000113** | NS | NS | NS | NS |
|  | loc_ss715637506 | A | G | NS | NS | NS | NS | NS | 0.001238* | NS | NS | NS | 0.001925* |
|  | loc_ss715637603 | A | G | NS | NS | NS | NS | NS | NS | NS | 0.000541** | 0.001000* | NS |

** significance threshold (5%), * suggestive threshold (10%)

^a^Williams 82

^b^Blacksburg, VA; Portageville, MO; Fayetteville, AR

^c^not significant

**Table S4.** Significant SNPs associated with the edamame Ala content

| Chromosome | SNP | WM82^a^ Allele | Alternative Allele | Environments^b^ | | | | | | | | | blue |
| --- | --- | --- | --- | --- | --- | --- | --- | --- | --- | --- | --- | --- | --- |
|  |  |  |  | Blacksburg, VA | | | Portageville, MO | | | Fayetteville, AR | | |  |
|  |  |  |  | 2018 | 2019 | combined | 2018 | 2019 | combined | 2018 | 2019 | combined |  |
| Chr 02 | loc_ss715582261 | A | C | NS | NS | 0.000377** | NS | NS | NS | NS | NS | NS | 0.001632* |
| Chr 06 | loc_ss715595114 | A | G | NS | NS | NS | NS | NS | NS | NS | 0.000408** | 0.000741 | NS |
| Chr 08 | loc_ss715602733 | T | C | NS | NS | 0.000626** | NS | NS | NS | NS | NS | NS | 0.000517** |
| Chr 10 | loc_ss715606089 | G | A | 0.001344* | NS | 0.000491** | NS | NS | NS | NS | NS | NS | NS |
| Chr 11 | loc_ss715611265 | A | G | NS | NS | NS | NS | NS | NS | NS | 0.001561* | 0.001704 | NS |
| Chr 13 | loc_ss715615623 | T | G | 0.001456* | NS | NS | NS | NS | NS | NS | NS | NS | 0.001911* |
|  | loc_ss715615635 | C | T | 0.001302* | NS | NS | NS | NS | NS | NS | NS | NS | 0.000665** |
|  | loc_ss715615637 | G | A | 0.001644* | NS | NS | NS | NS | NS | NS | NS | NS | 0.000853** |
|  | loc_ss715615638 | G | A | 0.000806** | NS | NS | NS | NS | NS | NS | NS | NS | 0.000296** |
| Chr 14 | loc_ss715617481 | A | C | 0.001088* | NS | NS | NS | NS | NS | NS | NS | NS | 0.001378* |
|  | loc_ss715617832 | T | G | NS^c^ | NS | 0.000029** | NS | NS | NS | NS | NS | NS | 0.000781** |
|  | loc_ss715617838 | G | A | NS | NS | 0.001902* | NS | NS | NS | NS | NS | NS | 0.000308** |
|  | loc_ss715617987 | C | T | NS | NS | 0.000074** | NS | NS | NS | NS | NS | NS | 0.000191** |
|  | loc_ss715619281 | T | C | NS | NS | NS | NS | NS | NS | NS | 0.000571** | 0.000319 | NS |
| Chr 17 | loc_ss715626474 | G | A | NS | NS | NS | NS | 0.001769* | 0.000761** | NS | NS | NS | NS |
|  | loc_ss715628043 | T | C | NS | NS | NS | NS | NS | 0.000293** | NS | NS | NS | 0.000082** |
|  | loc_ss715628044 | T | G | NS | NS | NS | NS | NS | 0.000042** | NS | NS | NS | 0.000068** |
|  | loc_ss715628048 | A | G | NS | NS | NS | NS | NS | 0.000811** | NS | NS | NS | 0.001110* |
|  | loc_ss715628050 | T | C | NS | NS | NS | NS | NS | 0.000829** | NS | NS | NS | 0.001077* |
|  | loc_ss715628063 | C | T | NS | NS | NS | 0.001406* | NS | 0.000378** | NS | NS | NS | 0.000231** |
|  | loc_ss715628064 | A | G | 0.001300* | NS | NS | 0.000958** | NS | NS | NS | NS | NS | 0.001458* |
|  | loc_ss715628065 | C | T | NS | NS | NS | 0.000695** | NS | 0.000164** | NS | NS | NS | 0.000134** |
|  | loc_ss715628067 | T | C | NS | NS | NS | 0.000183** | NS | 0.001281* | NS | NS | NS | 0.000960** |
|  | loc_ss715628069 | C | T | NS | NS | NS | 0.000386** | NS | NS | NS | NS | NS | 0.001014* |
| Chr 18 | loc_ss715632177 | G | A | NS | NS | NS | NS | NS | NS | 0.001416* | NS | 0.000599 | NS |

** significance threshold (5%), * suggestive threshold (10%)

^a^Williams 82

^b^Blacksburg, VA; Portageville, MO; Fayetteville, AR

^c^not significant

**Table S5.** Sucrose-related candidate genes and their descriptions within 10 kb flanking regions of significantly associated SNPs using Wm82.a2.v1.

| Chromosome | SNP | Gene ID in wm82.a2.v1 database | Functional annotation |
| --- | --- | --- | --- |
| Chr 3 | loc_ss715586179 | Glyma.03g200400 | HVA22 family |
| Chr 6 | loc_ss715594500 | Glyma.06g264600 | carbonic anhydrase |
|  | loc_ss715594504 | Glyma.06g265600 | leucine-rich repeat receptor-like protein kinase |
|  | loc_ss715595091 | Glyma.06g308200 | peptidyl-prolyl cis-trans isomerase |
| Chr 8 | loc_ss715599195 | Glyma.08g136500 | tetratricopeptide repeat (TPR)-like protein |
|  | loc_ss715599209 | Glyma.08g137500 | trehalose-6-phosphate synthase |
|  | loc_ss715599697 | Glyma.08g354800 | solute carrier family |
|  | loc_ss715602625 | Glyma.08g080000 | D-lactate dehydrogenase |
| Chr 9 | loc_ss715603160 | Glyma.09g021800 | plant invertase/pectin methylesterase inhibitor |
|  | loc_ss715604467 | Glyma.09g216200 | glycosyl transferases |
| Chr 10 | loc_ss715607940 | Glyma.10g268500 | fructose-bisphosphate aldolase |
|  | loc_ss715607973 | Glyma.10g270800 | trehalose-6-phosphate synthase |
| Chr 13 | loc_ss715607973 | Glyma.13g199400 | aspartyl proteases |
|  | loc_ss715616068 | Glyma.13g291400 | sterol regulatory element-binding protein |
|  | loc_ss715616116 | Glyma.13g297600 | endonuclease/exonuclease/phosphatase family |
|  | loc_ss715616737 | Glyma.13g068100 | alpha-mannosidase |
|  | loc_ss715617105 | Glyma.13g050200  Glyma.13g050300 | leucine-rich repeat receptor-like protein kinase  GATA-4/5/6 transcription factors |
|  | loc_ss715617106 |  |  |
|  | loc_ss715617107 |  |  |
|  | loc_ss715617181 | Glyma.13g046500 | glucosyltransferase |
| Chr 14 | loc_ss715618513 | Glyma.14g047500 | Endo-1,4-beta-glucanase |
|  | loc_ss715618537 | Glyma.14g048000 | subtilisin-like serine endopeptidase family protein |
|  | loc_ss715618543 | Glyma.14g048100 | leucine-rich repeat receptor-like protein kinase |
|  | loc_ss715619362 | Glyma.14g206100 | phototropic-responsive NPH3 family protein |
|  | loc_ss715619687 | Glyma.14g067500 | DVL family |
| Chr 16 | loc_ss715624465 | Glyma.16g159400 | leucine-rich repeat receptor-like protein kinase |
|  | loc_ss715625088 | Glyma.16g045100 | xyloglucan endo-transglycosylase |
| Chr 17 | loc_ss715626713 | Glyma.17g042400 | cytidine and deoxycytidylate deaminase zinc-binding |
|  | loc_ss715626717 | Glyma.17g042700 | transcription regulatory protein SNF5 |
| Chr 18 | loc_ss715629456 | Glyma.18g131300  Glyma.18g131500  Glyma.18g131600 | matrix metalloproteinase  endoplasmic reticulum metallopeptidase  peroxidase superfamily protein |
|  | loc_ss715629459 |  |  |
|  | loc_ss715629461 |  |  |
|  | loc_ss715629462 |  |  |
|  | loc_ss715629463 |  |  |
|  | loc_ss715629466 |  |  |
|  | loc_ss715629470 | Glyma.18g104700 | PIF1-like helicase |
|  | loc_ss715629479 | Glyma.18g131900 | multicopper oxidase |
|  | loc_ss715629481 | Glyma.18g132000 | peptidase dimerisation domain |
|  | loc_ss715629483 | Glyma.18g132100 | gibberellin regulated protein |
|  | loc_ss715629508 | Glyma.18g132400 | FAR1 DNA-binding domain |
|  | loc_ss715629516 | Glyma.18g132800 | plant protein of unknown function (DUF825) |
|  | loc_ss715631133 | Glyma.18g193600 | fructose-1,6-bisphosphatase |
| Chr 20 | loc_ss715637331 | Glyma.20g027800 | NAD dependent epimerase/dehydratase |
|  | loc_ss715637506 | Glyma.20g107200 | monooxygenase |
|  | loc_ss715637603 | Glyma.20g114300 | serine/threonine-protein kinase |

**Table S6.** Ala-related candidate genes and their descriptions within 10 kb flanking regions of significantly associated SNPs using Wm82.a2.v1.

| Chromosome | SNP | Gene ID in wm82.a2.v1 database | Functional annotation |
| --- | --- | --- | --- |
| Chr 2 | loc_ss715582261 | Glyma.02g200500 | AP2/B3-like transcriptional factor family protein |
| Chr 6 | loc_ss715595114 | Glyma.06g309900 | development and cell death related protein |
| Chr 8 | loc_ss715602733 | Glyma.08g104400 | programmed cell death related protein |
| Chr 10 | loc_ss715606089 | Glyma.10g032700 | transmembrane amino acid transporter protein |
| Chr 11 | loc_ss715611265 | Glyma.11g115000 | phosphatidylinositol transfer protein |
| Chr 13 | loc_ss715615623 | Glyma.13g245600 | cotton fiber expressed protein |
|  | loc_ss715615635 | Glyma.13g247100 | inositol-1,4-bisphosphate 1-phosphatase |
|  | loc_ss715615637 | Glyma.13g247200 | MYB-like DNA-binding protein |
|  | loc_ss715615638 | Glyma.13g247300 | aarF domain-containing kinase |
| Chr 14 | loc_ss715617481 | Glyma.14g015100 | cytochrome p450 cyp2 subfamily |
|  | loc_ss715617832 | Glyma.14g023000 | geranylgeranyl reductase |
|  | loc_ss715617838 | Glyma.14g023100 | senescence regulator |
|  | loc_ss715617987 | Glyma.14g028300 | glycerol-3-phosphate 1-O-acyltransferase |
|  | loc_ss715619281 | Glyma.14g201100 | O-methyltransferase |
| Chr 17 | loc_ss715626474 | Glyma.17g032400 | ATP synthase gamma-related protein |
|  | loc_ss715628043 | Glyma.17g070500 | S-adenosylmethionine decarboxylase |
|  | loc_ss715628044 | Glyma.17g070700 | GMP synthase |
|  | loc_ss715628048 | Glyma.17g071800 | zein-binding |
|  | loc_ss715628050 | Glyma.17g071900 | Ca^2+^/calmodulin-dependent protein phosphatase |
|  | loc_ss715628063 | Glyma.17g074200 | ubiquitin carboxyl-terminal hydrolase |
|  | loc_ss715628064 | Glyma.17g074300 | potassium channel tetramerization domain-containing protein |
|  | loc_ss715628065 | Glyma.17g074400 | fatty acid desaturase |
|  | loc_ss715628067 | Glyma.17g074800 | serine/threonine protein kinase |
|  | loc_ss715628069 | Glyma.17g075200 | Helix-loop-helix DNA-binding domain |
| Chr 18 | loc_ss715632177 | Glyma.18g269600 | O-methyltransferase |
